# Proteomics of Extracellular Vesicles: Update on Their Composition, Biological Roles and Potential Use as Diagnostic Tools in Atherosclerotic Cardiovascular Diseases

**DOI:** 10.3390/diagnostics10100843

**Published:** 2020-10-19

**Authors:** Alice Mallia, Erica Gianazza, Beatrice Zoanni, Maura Brioschi, Silvia Stella Barbieri, Cristina Banfi

**Affiliations:** Centro Cardiologico Monzino, IRCCS, 20138 Milano, Italy; alice.mallia@ccfm.it (A.M.); erica.gianazza@ccfm.it (E.G.); beatrice.zoanni@ccfm.it (B.Z.); maura.brioschi@ccfm.it (M.B.); silvia.barbieri@ccfm.it (S.S.B.)

**Keywords:** extracellular vesicles, exosomes, proteins, mass spectrometry, cardiovascular diseases, biomarkers

## Abstract

Extracellular vesicles (EVs) are lipid-bound vesicles released from cells under physiological and pathological conditions. Basing on biogenesis, dimension, content and route of secretion, they can be classified into exosomes, microvesicles (MVs) and apoptotic bodies. EVs have a key role as bioactive mediators in intercellular communication, but they are also involved in other physiological processes like immune response, blood coagulation, and tissue repair. The interest in studying EVs has increased over the years due to their involvement in several diseases, such as cardiovascular diseases (CVDs), and their potential role as biomarkers in diagnosis, therapy, and in drug delivery system development. Nowadays, the improvement of mass spectrometry (MS)-based techniques allows the characterization of the EV protein composition to deeply understand their role in several diseases. In this review, a critical overview is provided on the EV’s origin and physical properties, as well as their emerging functional role in both physiological and disease conditions, focusing attention on the role of exosomes in CVDs. The most important cardiac exosome proteomic studies will be discussed giving a qualitative and quantitative characterization of the exosomal proteins that could be used in future as new potential diagnostic markers or targets for specific therapies.

## 1. Introduction

Endogenous or internalized molecules involved in different and specific cellular mechanisms are compartmentalized into distinctly structured organelles; some of them can be released from the cell and play a role in physiopathological processes, embryofetal development, or metabolic regulation [1]. With the term extracellular vesicles (EVs) are defined lipid-bound vesicles secreted by cells in the extracellular space [2] both in physiologic conditions and under several stimuli such as hypoxia, stress, senescence, cell death, and particularly, inflammation [3]. Released vesicles are a key mediator of intercellular communication, transmitting signals that can interact with the recipient cell directly or by internalization [4]. EVs are produced by almost all cell types but their biogenesis and composition are influenced by the physiological cell state. EVs can be identified in all bodily fluids (e.g., blood, urine, cerebrospinal fluid) and can cross multiple biological barriers [5,6]. The main molecules enriching the vesicular space are lipids, lipid mediators such as eicosanoids, nucleic acids, and a wide range of proteins including cytokines, chemokines, lipid metabolism enzymes, and transmembrane proteins.

**Figure 1:**
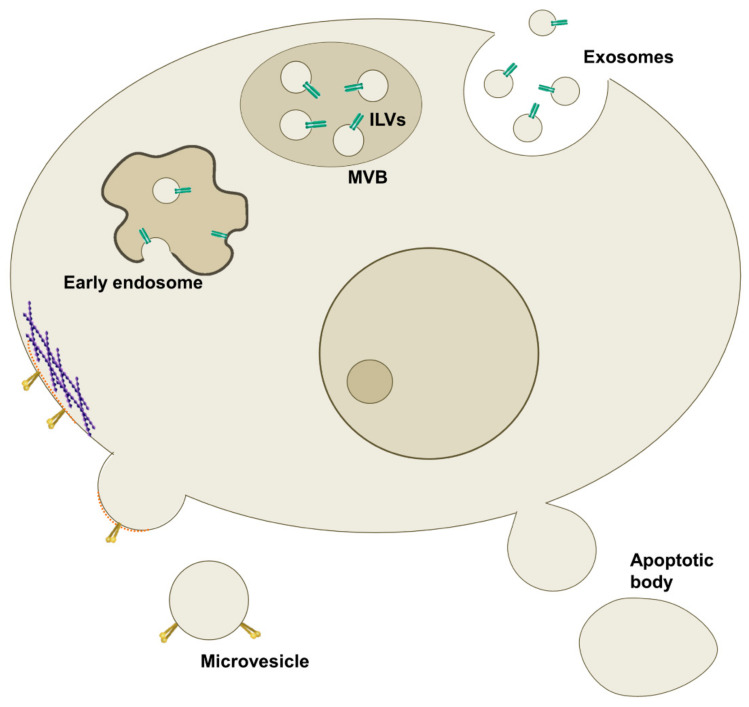
Different route of the release of extracellular vesicles (EVs). Schematic representation of the origin and release of EVs. Exosomes originate from the endosomal compartment as intraluminal vesicles (ILVs) in multivesicular bodies (MVBs), then released into the extracellular environment upon their fusion with the plasma membrane. Microvesicles (MVs) arise from the outward budding of the plasma membrane mediated by cytoskeletal remodelling and phospholipid rearrangements. Apoptotic bodies, instead, bleb consequently to cell death as a result of increased hydrostatic pressure after cell contraction.

A wide terminology has been used to identify EVs, but based on their biogenesis, cargo, biochemical composition, and release pathway, they can be classified into three main categories: exosomes, microvesicles (MVs), and apoptotic bodies [7] (Figure 1 and Table 1).

Exosomes, in particular, are enjoying great interest that has expanded over the past several years with rapidly growing literature on the topic, and this trend is even more pronounced for exosome proteomics publications.

Even though the review discusses the EVs’ origins and physical properties, as well as their emerging functional role in both physiological and disease conditions, attention is then focused on the role of exosomes in cardiovascular diseases (CVDs). Briefly, we will provide a critical overview on the most important exosome proteomic studies, dedicated to the qualitative and quantitative characterization of exosomal proteins that could represent a source of potential biomarkers for early disease diagnosis, prognosis, and response to a specific treatment.

### 1.1. Exosomes

Exosomes were first isolated in 1983 by Dr. Johnstone and colleagues studying the maturation of reticulocytes into erythrocytes [11]. They are cup-shaped homogeneous vesicles with a diameter of 30–150 nm and a flotation density of 1.08–1.19 g/mL. Exosomes originate from the endosomal network that is implicated in the sorting of intraluminal vesicles (ILVs) to their proper destination, such as lysosomes or extracellular environment. Specifically, they are formed within acidic endocytic organelles, the multivesicular bodies (MVBs), cytoplasmic structures containing multiple ILVs, and then released upon their fusion with the plasma membrane [12]. MVBs can either fuse with the plasma membrane to release ILVs extracellularly or alternatively with lysosomes to degrade their content [13]. However, the mechanisms that sort MVB to the lysosome or the plasma membrane are not yet clear, and it is not sure if the sorting between these two fates is dependent on individual ILVs or the entire MVB. Anyway, it has been shown that the inhibition of one pathway increases the other [14]. The endosomal system has a role in the quality control and degradation of membrane proteins. The majority of membrane proteins are rapidly recycled, either sorted to vesicles that proceed to late endosomes/MVB or to vesicles that recycle back to the plasma membrane. The late endosomes/MVB can fuse with plasma membranes or with lysosomes where their content is degraded becoming available for the cell again [14]. Recent studies also demonstrate that exosomes can be present in lysosomes where they are not degraded but released by lysosomal exocytosis [15].

Therefore, a deeper understanding of the mechanisms underlying the fates of MVBs is required, in order to have control of the complex sorting of MVBs.

Many mechanisms are involved in the recruitment of transmembrane and soluble molecules destined to exosomes. Transmembrane proteins that enrich exosomes’ membranes, such as glycosylphosphatidylinositol (GPI)-anchored proteins, are selected for their affinity for lipid domains and rafts, which are probably directly involved in ILV generation [16]. Cytosolic proteins, instead, are designated to be included in exosomes upon modifications, such as ubiquitylation or farnesylation. The sequestration of cytosolic proteins into ILVs can be mediated by co-sorting proteins, such as the chaperons heat shock protein 70 kDa (HSP70) and heat shock cognate 71kDa protein (HSC70) [17]. In addition to proteins, exosomes content is also characterized by nucleic acids. Specifically, mRNAs, miRNAs, and DNA sequences are found in circulating vesicles, but it is still not clear which are the mechanisms regulating their targeting and loading [18].

Exosomes formation is mediated by the activity of the endosomal sorting complex required for transport (ESCRT) components, which are four complexes (ESCRT-0/I/II/III), each playing a specific role in ILV assembly [19]. ESCRT-0 is a heterodimer composed by hepatocyte growth factor regulated tyrosine kinase substrate (HRS) and signal-transducing adaptor molecule (STAM) 1/2 proteins, both able to recognize ubiquitylated cargoes. HRS can further recruit clathrin thus facilitating the embedding of the cargo and the subsequent formation of ILVs. ESCRT-I and -II also contain a ubiquitin-interactive domain and work in concert with ESCRT-0 in the sorting of cargo with high avidity for ubiquitylated elements. Lastly, ESCRT-III leads to vesicle formation acting on membrane deformation and constriction of the neck of the membrane invagination [20]. Once the molecular content cargo is embedded within the nascent ILV, de-ubiquitylating enzymes (DUs) remove ubiquitin from included material. Contemporary, ATPase vacuolar protein sorting-associated protein 4 (VPS4) and co-factor vacuolar protein sorting-associated protein VTA1 homolog (VTA1) disassemble ESCRT machinery that can be further recycled [21].

Cargoes can be also targeted to nascent exosomes without the need of ubiquitylation and recognition by ESCRT-0/I/II. ESCRT-interacting protein ALIX (programmed cell death 6-interacting protein), together with ESCRT-III, is involved in the sorting of G protein-coupled receptor (GPCR) PAR1 (protease-activated receptor 1) or syndecans within ILVs independently from ubiquitin target [22]. Indeed, some studies pointed out that cells can correctly sort the cargo and release exosomes despite the depletion of ESCRT machinery components [23,24]. In these ESCRT independent mechanisms, exosomes formation could depend on sphingomyelinase enzymes, which convert sphingomyelin into ceramide [23] or can be induced by tetraspanin CD63, which is a protein involved in the formation of ILVs in melanosomes [25]. Ceramide may allow the generation of membrane subdomains that could induce spontaneous curvature or it can be metabolized into sphingosine 1-phosphate. This molecule can activate Gi-protein-couple sphingosine 1-phosphate receptor involved in the sorting of molecules into exosomal ILVs [26]. Furthermore, proteins of the tetraspanin family regulate the sorting of molecules addressed to nascent exosomes, such as CD63, CD81, CD82, and CD9. These proteins form clusters with other tetraspanins, membrane, and cytosolic proteins creating microdomains that will bud [25].

Given that ILV formation is regulated by the ESCRT machinery, the proteins of this complex together with their accessory ones (ALIX, TSG101, HSC70, and HSP90β) are considered specific exosomal marker proteins. Instead, it should be noted that tetraspanin proteins, expressed in the exosomes originating from an ESCRT independent pathway, cannot be considered as unequivocal markers of exosomes since they were found to also be expressed in MVs and apoptotic bodies [27].

MVB intracellular trafficking is regulated by several small GTPases Rab and soluble N-ethylmaleimide-sensitive fusion protein attachment protein receptor (SNARE) complexes [14]. Several studies showed that different Rab proteins are involved in the regulation of MVB trafficking and docking to the plasma membrane, but not their fusion with the membrane which is generally controlled in a calcium-dependent manner by SNARE proteins. Then, different physiological and pathological triggers supervised the release of exosomes. Rab and SNARE proteins also regulate lysosome fusion with MVBs.

Exosomes adopt a different role depending on their cellular origin. They are indeed able to modulate immune function, induce stem cell differentiation, inflammatory response, angiogenesis, lymphogenesis, cell migration, and proliferation. Furthermore, tumour-derived exosomes prime the distant organs to host cancerous cells facilitating the metastatic process [28].

Zhang et al. [29] studied exosome subclassification using an asymmetric flow field-flow fractionation (AF4) and demonstrated that they can be classified into a large and a small subpopulation, respectively, Exo-L (90–120 nm) and Exo-S (60–80 nm). Moreover, a distinct population of vesicles can be identified, known as non-membraneous nano-sized exosomes or exomers (35 nm), the most abundant group of particles released by cancer cells.

### 1.2. Microvesicles

MVs were initially described as subcellular material released from platelets in serum and plasma, and for this reason, also termed “platelet dust” [30]. Although this group of EVs was initially studied for its involvement in blood coagulation, it has been demonstrated to also have a role in cell-cell communication, even among cancer cells. Indeed, a subpopulation of MVs is known as oncosomes. Assuming that MVs are a heterogeneous family of vesicles, their size range is very broad, from 50 to 1000 nm or even more in the case of oncosomes [31,32].

MVs route formation is still not well understood. Biogenesis requires several molecular rearrangements of the plasma membrane, such as modification in lipids and proteins composition. Changes in the asymmetry of membrane phospholipids are guided by Ca^2+^-dependent enzymes, including aminophospholipid translocases (flippases and floppases), scramblases and calpain. These modifications lead to membrane curving and cytoskeleton remodelling, facilitating budding and MVs formation [33]. Moreover, MV formation requires cytoskeletal elements and their regulators, such as the RHO family of small GTPases and the RHO associated protein kinase (ROCK), involved in the modulation of actin dynamics in different populations of cancer cells [34].

Similarly to other EVs, MV content is characterized by lipids, proteins, and nucleic acids. Lipids and membrane components addressed to MVs are localized to sites of budding through their affinity to lipid rafts or by their anchoring to plasma membrane lipids, as in the case of oligomeric cytoplasmic proteins. Cytoplasmic elements require the association to inner plasma membrane anchors (palmitoylation, prenylation, myristoylation) and the assessment of high-order complexes that concentrate them in the site of budding [31]. It is still unclear how nucleic acids, generally present in MVs, are led to the side of bud, but an interesting study suggested the involvement of conserved zipcode RNA sequence motifs in the 3′ untranslated regions in mRNA targeting into MVs [35].

### 1.3. Apoptotic Bodies

The term “apoptotic body” was initially proposed by Kerr in 1972 [36]. The diameter of these EVs ranges between 500 and 4 μm, approximately the size of platelets. They are released as blebs which detach from a dying apoptotic cell as a result of increased hydrostatic pressure after cell contraction. Differently from exosomes and MVs, entire organelles can be carried inside apoptotic bodies, together with chromatin and a low amount of phosphorylated proteins. Furthermore, their membrane is enriched with phosphatidylserine on the external surface [27].

Apoptotic bodies are involved in different biological functions, including horizontal gene transfer, presentation of T cell epitopes after the uptake by phagocytic cells, and presentation of B cell autoantigens [37].

## 2. Emerging Role of Extracellular Vesicles

In addition to their heterogeneity, which affects size, luminal content, and membranes, EV composition is subjected to change upon environmental stimuli such as thermal and oxidative stress [38,39]. EVs may target cells by the transfer of proteins, nucleic acids, lipids, and metabolites after the recognition and binding to specific membrane receptors. They have been demonstrated to have roles in intercellular signalling, in pathological conditions ranging from the regulation of immune responses to cancer metastasis and CVDs where the composition and quantity of EVs are reported to change [40,41]. Functions of EVs have been also reported in neurodegenerative and autoimmune diseases. Moreover, they have been implicated in physiological processes like blood coagulation, tissue repair and synaptic plasticity [12].

**Figure 2:**
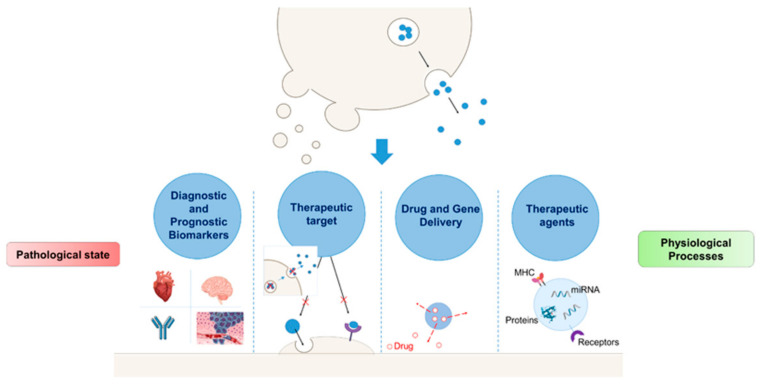
Potential applications of extracellular vesicles (EVs). Involvement of EVs in the pathogenesis and progression of several diseases. Based on these effects, EVs can be translated into therapeutic targets and useful disease biomarkers. Further, their role in the maintenance of fundamental physiological processes like blood coagulation, stem cell plasticity, and tissue repair supports their potential application as therapeutic agents. Finally, EVs can transfer their contents to target cells, thus providing their possible use as a drug delivery system.

Contemporary, there is an increasing interest in the use of EVs in diagnostics, therapeutics, and drug delivery (Figure 2).

Focusing on their luminal load, EVs are suitable candidates as a source of biomarkers as their content varies according to the cell of origin and the disease status. For this reason, they can be more specific markers than other molecules. Furthermore, unlike circulating plasma biomarkers, the conveyed molecules are preserved from enzymatic and hydrolytic degradation in the extracellular environment [12]. The prognostic and diagnostic potential of targeting EVs as biomarkers can be exploited for several pathologies including CVDs as reviewed by Boulanger et al. [40]. Further, containing drug metabolizing enzymes, EVs have also been described as a potential tool for the characterization of drug metabolism and drug exposure variability [42,43].

The biological role of EVs depends on their origin and composition. Exosomes, which have been characterized mostly in cancer and immune cells, are implicated in the transfer of oncogene receptors, HIV particles, mRNA, and miRNA [37]. Thanks to that, the research mainly focused on the study of exosomes as tumour diagnostic biomarkers. They are now potential tools for the diagnosis of glioblastoma, ovarian, lung, and breast cancer [44]. Exosomes are also ideal candidates for gene therapy [45], they indeed possess immunomodulatory activities and can act as antigen-presenting cells. MVs, instead, have been characterized predominantly as products from blood cells although their presence in serum suggests they could also be originated from other cells. Endothelial MVs, present in the blood, can be used as biomarkers of vascular damage as they underline an inflammatory condition, endothelial injury, and endothelial dysfunction [46]. At the same time, MVs derived from platelets can be associated with CVD progression and autoimmune diseases as type II diabetes [37].

EVs’ endogenous properties, which are fundamental for the maintaining of a physiological state, in some contexts can lead to EV involvement in disease by inducing unwanted effects. For this reason, the inhibition of EV production and release may result in being advantageous both as a research tool and therapeutic approach. EV effects in disease state can be reduced by preventing biogenesis [47], releasing [48], up-taking, and target cell response [49]. However, the complexity of EVs’ biogenesis and releasing complicates the development of a single selective drug able to inhibit EV trafficking and to identify EV subpopulation associated with a specific compromised cell or tissue. Nevertheless, some drugs already approved for other clinical indications revealed themselves as promising, even if their side effects, especially those not related to their effect on EVs, should be considered [50]. Further, healthy tissues can also be affected by the inhibition of EV trafficking and this may lead to other potential secondary effects [51].

As EVs contribute to the modulation of normal physiological processes, they have been investigated as therapeutic agents for the development of anticancer vaccines [52] and, thanks to their immunomodulatory properties, for the treatment of autoimmune and neurodegenerative diseases [49]. It has been pointed out that EVs derived from stem cells, supporting the healing of injured tissues, could be applied as a potential therapeutic alternative in the treatment of myocardial infarction (MI) [53] and ischemia [54].

Indeed, it is well known that mesenchymal stem cells exert their therapeutic effect due the ability to secrete proregenerating factors, creating a local microenvironment favourable for the healing process [55]. In this context, many studies have been focused on umbilical cord stem cells for their ability in releasing exosomes with proved regenerating activities [56,57,58]. Recent papers suggest that isolated stem cell-secreted nanoparticles carry several important molecules involved in cell-cell crosstalk and offer many advantages for the use in the clinical setting [59].

In comparison with treatment with stem cells themselves; exosomes show a high level of safety, in terms of induction of immunological reactions. Indeed, from a clinical point of view, the use of stem cells in regenerative medicine suffers from safety problems linked, for example, to the use of animal serum for their growth. As an alternative, the use of human allogenic platelet lysate, safer and quality controlled, has been proposed [60]. Marrazzo et al., recently demonstrated the effectiveness of the treatment with platelet lysate on dental pulp stem cells for bone and cartilage regeneration [61], promoting their osteogenic and chondrogenic differentiation.

Finally, thanks to their favourable properties such as increased stability, long-circulating half-life, biocompatibility, low immunogenicity, and toxicity, EVs, especially exosomes and their bioengineered products, are ideal for the delivery of drugs, proteins, miRNA and siRNA, and other molecules that would otherwise be rapidly degraded. The use of exosomes as potential delivery systems revealed an effective, in terms of drug distribution and toxicity, method for the treatment of different pathologies including cancer and neurological diseases [62].

## 3. Proteomic Methods

Nowadays, mass spectrometry (MS) is a fundamental technique for the identification and characterization of the protein content of EVs, and it is applied to study EVs in several diseases such as CVDs, cancer, and neurodegenerative diseases.

As described before, EVs can be released from many different cell types and they can be isolated from biological fluids, cell culture-conditioned medium [63,64], and dissociated tissues [65], even if sometimes it is not easy to determine the nature of recovered EVs because some intracellular vesicles can be released during the tissue dissociation procedure.

A typical workflow of MS-based proteomic studies of EVs involves several steps, among which the isolation of EVs from specific biofluids, the extraction of EV proteins using detergent or non-detergent lysis buffers, the separation of the extracted proteins and digestion before the MS analysis. In particular, the extracted EV proteins can be separated by gel electrophoresis and in-gel digestion, otherwise they can be directly digested and then obtained peptides are fractionated by liquid chromatography (LC) before the MS analysis.

Although a large variety of methods and technologies for EV isolation and detection are available, there is no a single specific and rigorous procedure for successful exosome purification and characterization in complex biological samples that can be used as a reference method. Usually, more methodologies are employed in combination to allow the characterization of a pure exosome population. For this reason, the review summarizes the most commonly applied methods for proteomic EV preparation and analysis with their potentials and limitations, also describing the recent advances in improving the isolation sensitivity of exosomes.

### 3.1. Sample Collection, Storage and Processing

Appropriate sample storage and processing is crucial to avoid significant changes of the EV amount and composition, which consequently lead to a pre-analytical variance and biased results. Standardized protocols of biological fluid handling and storage are required to guarantee accurate and comparable measurements of EVs. Therefore, some general important indications have been consolidated by previous studies and universally adopted nowadays [66,67]. Samples should be processed immediately after the collection (e.g., within 2 h after blood collection to prevent an increase in EV levels [68]), repeating freezing-thawing cycles should be avoided, and the sample storage should be at −80 °C until EV isolation. Siliconized tubes are recommended for storage because they have very low surface adhesion providing maximum EV recovery, and phosphate buffered saline (PBS) is usually used for sample resuspension and dilution. Anyway, standardization of the processes from sample collection to storage should be well established based on the type of body fluid, considering that the different characteristics of fluids and their preparation protocols (e.g., any used anticoagulants and centrifugations) could affect EV recovery [67]. For example, the effects of a single freeze-thaw cycle and storage of up to 1 year at −80 °C are irrelevant on the EV concentration and size in plasma, whereas they could generate a moderate variability in urine and saliva, even if changes in EV composition and function may not be excluded [67,69]. Despite extensive studies on EVs, until now the reasons of this variability have not been clearly explained, but it is certainly important to define, characterize, and standardize the pre-analytical conditions such as freezing and storage in comparative studies on EVs to achieve a consistent and effective measurement and avoid systematic errors [67].

EVs can be preserved by freezing or lyophilization for long-term storage. Anyway, Bari et al. demonstrated that both approaches can reduce the bioactivity of EVs by compromising their integrity [70]. Lyophilization (freeze drying) is commonly applied to obtain easy storage with long-term stability but it can cause freezing and drying stresses that can alter the stability of the biomolecules structure within the EVs. For this reason, a cryoprotectant is needed to stabilize the biological membrane and protect the EV protein content during lyophilization as well as to inhibit the vesicle aggregation following dehydration. Cryoprotectant compounds have different formulations containing one or more substances called “excipients”, such as buffer/pH adjusters, stabilizing agents, or tonicity modifiers [71]. For example, mannitol was used as an effective cryoprotectant in maintaining EV integrity and preventing aggregation during the lyophilization process [70]. Furthermore, other sugar stabilisers have been successfully used showing that EVs were stable upon freezing and their interaction with target cells was not compromised [72].

The maintenance of the integrity of EV membrane phospholipids is crucial to protect internal content, which must reach the surrounding tissues without degradation. Therefore, lyophilisation of EVs provides easy handling at room temperature encouraging the use of lyophilisation as future EV storage approach without compromising their biological cargoes [73]. Moreover, when platelet-derived EVs are investigated, it is important to prevent platelet activation or degradation during the collection and handling by physical forces, thereby avoiding the release of EVs [66].

Human plasma, which contains EVs both from platelets and erythrocytes, is the most commonly studied fluid, but in the last years there has been great interest to also study other fluids, such as urine, saliva, amniotic fluid, and cerebrospinal fluid [66]. It is known that saliva is enriched with exosomes, responsible for the transport of several messengers between different oral cell types representing a defence from microbial infection, given their activity in intercellular transportation and modulation of cell-mediated immunity. The control of the oral microbiota, and more generally the systemic microbial homeostasis, is indeed an important issue influencing the diagnosis and progression of complex diseases [74].

It is also possible to investigate EVs from a single cell type, which can be easily obtained by collecting conditioned medium from cells cultured with or without a specific stimulus.

It is equally important to also note that many factors can affect the process of vesiculation, among which are age, gender, body mass index, medication use, physical activity [75], pregnancy [76], fasting, or postprandial status [77], thus a careful selection of the case histories in EV studies is essential both in discovery and diagnostic research [66].

### 3.2. Isolation Methods for MS-Based Proteomic Studies of Extracellular Vesicles

An isolation step of EVs is essential for proteomic studies, because EVs have a lower concentration in biological fluids than the other abundant components, such as albumin and lipoproteins, which could interfere with the proteomic characterization of EVs. The isolation methods can exploit the physical characteristics of EVs (e.g., size and density) or their chemical properties. Different methods are available for EV isolation, including differential ultracentrifugation, filtration, immunoaffinity, and microfluidics techniques. They can be chosen according to the required degree of EV purity and concentration, and they may be employed either individually or in combination. Each method has both advantages and disadvantages (Table 2), thus it is difficult to determine the reference method. Moreover, each type of biofluid has different considerations to be noted regarding the centrifugation.

#### 3.2.1. Differential Ultracentrifugation

Differential ultracentrifugation is the most commonly used method for the isolation of EVs from biological fluids or cell culture media [78]. A dilution of biological fluids with PBS before centrifugation can increase the EV purity and recovery by decreasing the viscosity, even if this procedure is not always required for each fluid [79]. A consecutive intensification of centrifugation forces and times allows isolation of smaller particles from larger ones according to their sedimentation rates. After sample dilution, dead cells and cell debris are removed by one or more centrifugation steps at 1000–3000× *g* for few minutes at 4 °C. Then, MVs are isolated from exosomes by higher speed centrifugation of the supernatant at 10,000–20,000× *g* for about 30 min at 4 °C. At this point, a supernatant filtration is sometimes carried out to remove particles larger than 200 nm. Finally, the recovery of exosomes is performed by ultracentrifugation at 100,000–200,000× *g* for hours [80,81,82] and the pellet is washed by resuspension with PBS and centrifuged again to remove contaminants and improve the purity.

For samples with high viscosity, higher centrifugation speed and time are required. Therefore, the efficiency of the EV isolation is also dependent on multiple parameters that can influence the type, quantity, and quality of the EVs isolated by differential ultracentrifugation, but their simultaneous control is difficult [83].

Moreover, to accurately estimate the protein amount of the exosomal pellet from cell culture media, the culture medium must be completely removed from the pellet because it includes amino acids and phenol red that can interfere. It has been also suggested that albumin and other proteins or metabolites found in the foetal bovine serum used in cell culture experiments can influence experimental results. Therefore, using several depletion methods, serum-free medium, or EV-free serum are often used to minimize the contaminations and collect exosomes from cell culture media [84,85].

#### 3.2.2. Density-Gradient Ultracentrifugation

EV pellets are often contaminated by other high abundant molecules (e.g., lipoproteins, protein aggregates, soluble proteins) or proteins that bind non-specifically to the exosomes and can interfere with further MS analysis. A density gradient flotation, such as the sucrose gradient [86] or the iodixanol (OptiPrepTM) velocity gradient [87,88], can be applied to the differential ultracentrifugation protocol to separate large protein aggregates from exosomes [89]. Indeed, even if the density of MVs remains unclear, the density of exosomes is about 1.08–1.19 g/mL [90]. Upon elevated centrifugal force EVs migrate through the surrounding medium, and separate based on their buoyant density, resulting in further purification of EVs from contaminating proteins. Sucrose is broadly used but it has high viscosity, and is hypertonic, thus precluding its use in the separation of osmotically sensitive particles. Therefore, an iodixanol gradient (5%–40%) can be used instead of sucrose to preserve the size of EVs in the gradient forming iso-osmotic solutions over a wide range of densities [91].

Additional strategies can be also applied for different biological fluids to increase EV purity. For example, in urine samples uromodulin forms a network that leads to trapping of exosomes during centrifugation, thus a treatment of the exosome pellets with dithiothreitol (DTT) [92] or 3-((3-cholamidopropyl)dimethylammonio)-1-propanesulfonic (CHAPS) [93] can inhibit the aggregation allowing the release of exosomes [94]. However, it is important to consider that DTT is a strong reducing agent that causes a remodelling of exosomal proteins thus modifying their biological activity. In contrast, CHAPS is a mild detergent used to solubilise proteins, and it has been demonstrated that it does not influence the EV morphology or exosomal marker distribution preserving their biological function [93]. Another example of contaminants are lipoproteins, which are frequently found in EV preparations from plasma. Some research groups usually perform additional washing steps of the exosomal pellet with KBr to solubilise lipoproteins, and remove them from the plasma [95].

#### 3.2.3. Size-Based Isolation

Filtration and size exclusion chromatography (SEC) are size-based isolation methods that can be applied alone or in combination with other techniques to isolate EVs from biological fluids and cell lines.

Samples can be filtered through a membrane with a specific pore size by pressure or centrifugation [96,97]. Tangential flow filtration (TFF) was applied for a highly efficient isolation of EVs [98]. In TFF the fluid flows tangentially across the surface, avoiding the filter clogging. Other filtration methods, such as field flow fractionation (FFF) [99] and AF4 [29], were successfully used to separate exosomes by size. Recently, a “hydrostatic filtration dialysis” method was also developed to enrich human urinary EVs, and characterize the integral transmembrane proteins present [100]. Anyway, filtration is often used in combination with other methods for EV isolation, such as differential ultracentrifugation, to concentrate the sample more, and remove interfering components. As mentioned before, a filtration of the supernatant from ultracentrifugation can be achieved using a 0.22 µm filter device to remove components with a diameter larger than 200 nm [101].

Size exclusion chromatography is another type of size-based isolation strategy that consists of moving the sample through a column packed with heterogeneous polymeric beads (e.g., Sepharose and Sephadex) with diverse pore size [78]. SEC separates proteins in solution based on their sizes, thus larger sized species are eluted earlier than the smaller molecules of interest that can penetrate all the stationary phase pore system and elute later. SEC ensures exosomes with high purity that can be used to measure levels of potential biomarkers in small-volume clinical samples [69]. SEC is often used in combination with other techniques to ameliorate the separation procedure, and sometimes it can be applied as the last step of the differential ultracentrifugation [102]. A commercial SEC column, known as qEV, is also available to obtain rapid and effective EV isolation, exosomes with higher purity than those obtained using differential ultracentrifugation or precipitation methods, but also reliable and reproducible results. For that reason, qEV is now considered the most used enrichment method that allows having homogenous exosomes in terms of size, morphology, and protein composition [103].

#### 3.2.4. Immunoaffinity Isolation

Highly selective separation of exosomes can be also obtained using specific antibodies. The affinity-based isolation of exosomes is possible through the interaction between the surface markers of EVs and the antibody that is typically bound to either a device surface or a magnetic bead. Several proteins, such as HSPs, cytoplasmic proteins (e.g., actin, tubulin, Rab proteins, and annexins), and tetraspanins, are common to all EVs, and often used as markers to isolate them [94,104,105]. Recently, Lim et al. reported the successful use of antibody cocktail-conjugated magnetic nanowires to isolate exosomes from plasma of breast and lung cancer patients, resulting in higher efficiency in exosome isolation with a greater yield and purity compared to other conventional methods [106].

Certainly, immuno-based isolation can be also applied to isolate specific EV subpopulations using specific protein markers not common to all vesicles and correlated to a definite function [88,107,108]. Moreover, specific synthetic peptides, such as Vn and heparin, have been also used as EV-binding molecules to isolate EVs. Vn with a specific affinity to HSPs has been used to select EVs expressing these proteins from breast cancer cell lines and patients [109], and its suitability for global proteomics and high-throughput biomarker discovery has also been evaluated [110]. Heparin has been used to isolate EVs, in which heparan sulphate proteoglycans are expressed as cell surface receptors, from conditioned cell culture media as well as from plasma samples. Heparin-coated beads were used after ultrafiltration, for example, to isolate EVs released from normal and cancer cells, and from human blood plasma, showing the expression of the specific EV marker ALIX, a multifunctional protein frequently used as a marker of EVs, and lower level of protein contamination [111].

However, an affinity for the lipid membrane structures of EVs is often applied to facilitate plasma EV extraction. The enrichment of phosphopeptides on the lipid bilayer of EVs by titanium dioxide (TiO_2_) affinity chromatography allowed a rapid and efficient isolation of high-quality exosomes from human serum of pancreatic cancer patients and healthy subjects [112]. Besides, EVs can be purified based on their membrane phospholipid composition, in particular phosphatidylserines and GM1 gangliosides. EVs have been successfully isolated by using Tim4 protein, which binds the phosphatidylserine expressed on the surface of EVs and then easily releases the EVs by adding Ca^2+^ chelators. Tim4-purified EV preparations showed higher purity than those obtained using conventional methods, providing the possibility to detect and quantify more EV-specific proteins by MS [113].

#### 3.2.5. Polymer-Induced Precipitation

The polymeric precipitation technique is based on the use of polymers such as polyethylene glycol (PEG) to entrap EVs, providing a rapid isolation of EVs from biological samples or culture media [114,115]. Several commercial isolation kits have been produced for the application of PEG solutions to isolate exosomes, such as the most commonly used Exo-spin exosome purification kit, ExoQuick exosome precipitation solution and Invitrogen total exosome isolation kit [116]. In order to remove the contaminating proteins and separate EVs [117], polymer-induced precipitation is often supported by pre- and post-isolation steps, such as SEC or the use of Sephadex G-25 spin columns which retains the polymer from precipitated EVs [118,119].

### 3.3. MS-Based Strategies for the Study of Extracellular Vesicle Proteome

Actually, the majority of the proteomic studies of EVs are performed using a bottom-up MS approach, which involves the extraction of proteins from the isolated EVs, the separation of the extracted proteins, and their digestion before MS analysis [78]. On the other hand, top-down proteomics has been less applied in EV research, mostly in exosome studies (e.g., [120]), even if it allows sequencing of intact proteins and their proteoforms without the need of proteolytic digestion before MS measurement. Indeed, despite the improvements in top-down proteomics, the high costs of the instrumentation, the low-efficiency of the dissociation techniques, and the complicated mass spectra lead to some limitations in the application of top-down proteomics to study specific modifications of intact circulating EVs proteins and characterize them [121].

Proteins are usually extracted using a detergent (e.g., sodium dodecyl sulphate (SDS)) or non-detergent (e.g., 8 M urea) lysis buffer. TRIzol reagent has been also suggested for the extraction of proteins from EVs because of its extraction capacity similar to the commonly used Laemmli reagent and its ability to simultaneously isolate DNA/RNA and proteins from the same EV sample, thus reducing the processing time [122].

Specific extraction protocols have been also proposed for the study of sub-populations of proteins in the EVs, such as membrane proteins [100], providing a targeted isolation of proteins and facilitating the detection of potential biomarkers or drug targets in EVs.

Filter aided sample preparation approach (FASP), a proteomic method for buffer exchange and protein digestion before MS analysis, and the related multiple enzyme digestion (MED) FASP have been successfully used in several EV studies, providing easy and efficient protein processing and minimal sample loss [123,124].

An additional separation step of the extracted EV proteins is usually performed before MS analysis using either gel electrophoresis [125] or LC [126,127] to facilitate proteomic analysis removing the contaminants in the sample. Moreover, specific separation methods for the study of post-translational modifications of EV proteins have also been applied in many proteomic studies. As an example, the prolonged ultracentrifugation-electrostatic repulsion-hydrophilic interaction chromatography (PUC-ERLIC) was applied to study EV-enriched glycoproteins [128], while the immobilized metal ion affinity chromatography (IMAC) was employed to enrich phosphopeptides derived from the EV proteins’ digestion [129]. A few years ago, Wu et al. also described a rapid isolation method known as EVTRAP for the highly efficient purification of EVs and further EV phosphoproteome analysis using polyMAC-based phosphopeptide enrichment [130].

The MS analysis in EV proteomic studies is usually performed by data-dependent acquisition (DDA) [131] or data-independent acquisition (DIA) such as SWATH (sequential window acquisition of all theoretical fragment ion) [81], MS^E^ [92], and multiplexed MS/MS.

Quantitative MS based on label and label-free approaches has been applied to study EV proteome in several diseases. Label-free approaches are simple and cost-efficient techniques that have been extensively used in EVs research [132,133,134], while label-based approaches include a metabolic or chemical labelling which provides more accurate protein quantification [127,135].

MS is an effective approach for identifying potential EV-derived biomarkers or targets for drug treatments in pathological conditions. A DIA-MS-based diagnostic method was recently described to study EV protein and phosphoprotein biomarkers in liquid biopsies of patients with colorectal cancer and the DIA-MS quantifications were then confirmed by a targeted approach known as parallel reaction monitoring-mass spectrometry (PRM-MS) [136]. A quantitative proteomic analysis based on a stable isotopic labelling method using isobaric tags for relative and absolute quantitation (iTRAQ) of urinary EV proteins was performed to discover potential biomarkers for prostate cancer diagnosis, and then the candidate proteins were verified using multiple reaction monitoring (MRM) MS [137]. Similarly, iTRAQ labelling was also used to quantify human seminal EV proteins [138]. A stable isotope labelling with amino acids in cell culture (SILAC) combined with high-resolution MS was also applied to perform a comprehensive analysis of proteins in microsatellite unstable colorectal cancer-derived EVs [139]. Moreover, a PROMIS-Quan (PROteomics of MIcroparticles using Super-SILAC Quantification), which is based on SILAC quantification, was described by Harel et al. for valid plasma microparticle extraction, followed by SILAC-based relative and absolute quantification [140].

Targeted quantitative MS was also used to evaluate the purity of EV preparations. For example, MRM was applied using QconCATs as internal standards for the quantification of EV and non-EV proteins, thus comparing the purity of EVs preparations obtained from human serum by two different isolation protocols [141].

## 4. Exosomes in Cardiovascular Diseases

The exosomes contain proteins that are involved in cellular communication and their important role has been investigated in several biological fluids and cell types. Great interest has been placed in the application of exosome research for the discovery of biomarkers for disease and therapeutics [142]. Because of the exosome ability to cross the blood brain barrier, their analysis is minimally invasive in the context of fluid biopsy.

Cardiac exosome research is a promising field of research that has gained attention in recent years to elucidate the intercellular communication in the heart and discover potential biomarkers of CVDs. Exosomes have a central role in intercellular communication in the heart, which is important in the maintenance of physiological cardiac homeostasis and the stress response [143]. They are involved in multiple physiological and pathological cardiovascular processes, and they have a key role in regulating CVD progression through the transport of signal molecules [144].

Numerous studies have made it possible to strengthen the knowledge of the exosome proteome in cancers and immunological diseases (e.g., [145,146,147]), while the characterization of the cardiovascular exosome proteome is still in development. Several methodologies are available to isolate exosome from other factors such as cell fragments, larger vesicles, or soluble molecules, but the choice of analytical method for exosome purification is critical due to the differences in the protein identifications that have been reported in many studies (e.g., [89,148]). This variability in protein identification suggests the importance of a high exosome purification and the presence of a heterogenous population of proteins inside the exosomes that is dependent on the cell type or biofluid of origin. Therefore, different exosome populations with both distinct protein and RNA composition are constitutively released by cells or in response to a variety of physiological or pathological stimuli.

Currently, there are no complete and specific proteome profiles of the exosomes in diseases that could be used for diagnosis based on exosomes alone, and there is also no technology for the detection and analysis in clinical practice that is rapid and high-throughput to obtain accurate results with low inter-laboratory variability and high quality control [142]. However, many efforts have been made so far in the study of exosome profiles extending the knowledge of the protein content and implementing the technologies for an increasingly effective purification.

Several standard proteomic approaches have been used in the proteomic analysis of exosomes, and in particular, two-dimensional gel electrophoresis (2-DE) and LC-MS/MS or MALDI-TOF/TOF MS are the mostly applied. More than a thousand different proteins have been isolated and the most common exosomal proteins are tetraspanins (CD9, CD63 and CD81), membrane transporters, fusion proteins (annexins, GTPases, and flotillin), heat shock proteins, ESCRT proteins, and multivesicular body synthesis proteins (ALIX and TSG101). These proteins are generally used as marker proteins for exosomes. Moreover, a recently study on the exosomes enriched from human serum identified with high confidence by MALDI-MS analysis the platelet factor 4 as a novel exosome marker that can be used as an alternative tool for confirming the exosome presence [149].

CVDs include several conditions affecting the heart or blood vessels and it has been already demonstrated in previous studies that the number and content of EVs can be different based on the disease state thus making EVs a source of potential biomarkers. As mentioned above, exosomes are messengers exchanging signal molecules among cardiomyocytes, fibroblasts, endothelial cells (EC), and the immune system, leading to the regulation of cardiac apoptosis, hypertrophy, angiogenesis, fibrosis, inflammation, and immune response. Cardiac exosomes derive from different cell sources and can exert beneficial effects or adverse effects in the various pathological processes [144].

### 4.1. Exosomes Derived from Cardiomyocytes

The exosomes present in cardiomyocytes are known as “cardiosomes” and they are involved in regulating several processes such as angiogenesis and also in transferring nucleic acids to target cells [150]. The exosome population derived from cardiomyocytes is not homogenous and a degree of heterogeneity in the exosome appearance and surface antigens is present leading to differences in cargo and target cells [150].

HSPs are enriched in cardiac exosomes and they show a key role in cellular survival and protection to multiple stressful events. HSP20 is an important member of the HSP family and is present in cardiomyocyte-derived circulating exosomes, promoting the formation of myocardial neovascularization and blocking tissue necrosis factor α (TNF-α) and interleukin 1β (IL-1β) to reduce the risk of MI [151]. HSP20 is released in physiological conditions, but its secretion is increased in MI and under stress conditions such as ischemia/reperfusion [152]. Similarly, HSP70 is expressed in the cardiac exosomes and it is involved in the regulation of cardiomyocyte growth and survival under stress stimuli. Cardiac exosomes are also enriched for HSP60 which has an important role under physiological conditions, but it also plays a regulatory role in the progression of pathological conditions such as heart failure and atherosclerosis [153]. HSP60 in the exosomes is found to be attached to the exosome membrane, and after its release by cardiomyocytes via exosomes, extracellular HSP60 is generally considered as a damaging signal to the surrounding cardiac myocytes because it stimulates the release of TNF-α and IL-6 from cardiomyocytes and increases the expression of Toll-like receptor (TLR) 4 causing cardiac myocyte apoptosis [154]. Malik et al. investigated the stability of exosomes isolated from adult rat cardiac myocytes under physiological and pathological conditions, and performed a LC-MS/MS analysis of their protein content after two different treatments, mild hypoxia and ethanol [154]. They demonstrated that exosomal protein content differs depending on the stimulus and that HSP60 is stable within the exosome and not released under a range of physiological conditions. Thus, if HSP60 is present inside the exosomes, this should prevent its toxicity to cardiomyocytes.

The exosomes can be released from all cells in the cardiovascular system and their release and cargos can be modulated under stressed conditions such as inflammation or hypoxia, improving or impairing the heart function [155]. Indeed, it has been demonstrated that the secretion of exosomes by cardiac myocytes is significantly increased under hypoxic conditions and also their content is modified [156]. Yu et al. investigated the direct effects of hypoxia on TNF-α expression of cardiomyocytes and for the first time they observed higher TNF-α expression and hypoxia inducible factor-1α (HIF-1α) activation in primary cultured cardiomyocytes under hypoxia, specifically exosomes are involved in the autocrine effects of TNF-α in hypoxic cardiomyocytes [157]. HIF-1α induces the transcriptional activation of TNF-α in response to hypoxia and TNF-α is secreted by the cardiomyocytes via exosomes to other healthy cells leading to increased inflammatory reaction and apoptosis. TNF-α is not constitutively expressed in normal hearts, while in acute myocardial infarction (AMI) the elevated TNF-α expression in cardiomyocytes is damaging and contributes to cardiac remodelling.

Exosomes secreted by cardiac cells can be internalized by neighbouring cells or released into the bodily fluids, allowing the use of exosome proteins as potential biomarkers of a pathological state considering that their expression levels can be varied depending on the disease.

In a comparative proteomic profiling study on plasma EVs obtained from patients with MI and patients with noninfarcted stable angina, Cheow et al. demonstrated that the harvested EVs showed morphology and proteomic characteristics consistent with that of exosomes and identified a novel biomarker panel of six overexpressed proteins with diagnostic and therapeutic potential for patients with coronary artery diseases (CADs) [158]. This panel included proteins involved in post-infarct pathways of complement activation, lipoprotein metabolism and platelet activation.

A qualitative label-free shotgun proteomics by nanoLC-MS/MS was applied to study exosomes extracted from plasma and pericardial fluids in patients with AMI undergoing coronary artery bypass surgery and patients undergoing surgery either for aortic root aneurysm or valvular disease (control group) [159]. The authors demonstrated that pericardial fluid from patients with MI induces epicardial epithelial-to-mesenchymal transition (EMT) which is involved in cardiac tissue repair following injury. Exosomes were detected both in pericardial fluids and plasma and, in particular, 61 and 65 exosome-derived proteins were identified in pericardial fluids from AMI patients and controls, respectively, whereas 68 and 56 exosome-derived proteins were identified in plasma from AMI patients and controls, respectively. Proteins involved in transcriptional regulation pathways were specifically identified in patients with AMI, and, among these proteins, the authors identified clusterin as a highly enriched component of pericardial fluid-derived exosomes from patients. It has been shown that clusterin induces EMT in epicardial cells, stimulates neoangiogenesis, and improves cardiac function, in addition to being involved in inflammation and immune response. Many studies have highlighted the involvement of clusterin in all stages of atherosclerosis suggesting its potential role as biomarker of atherosclerosis lesions and CVDs [160,161].

Over the years, numerous studies have also demonstrated that endogenous plasma exosomes can transfer signals to the heart providing protective effects against ischemia and reperfusion injury. Exosomes isolated from the blood of adult rats and human volunteers displayed cardioprotective properties against ischemia and reperfusion both in vitro and in vivo [162]. This cardioprotection was mediated by HSP70 exposed on the surface of exosomes, which stimulated TLR4 receptor and activated the mitogen-activated protein kinase (MAPK)/extracellular signal-regulated kinase (ERK) 1/2 signal pathway, leading to phosphorylation of the cardioprotective HSP27 in cardiomyocytes.

Abdominal aortic aneurysm is a vascular pathology with high morbidity and mortality rates that usually has no symptoms making its detection very difficult. Martinez-Pinna et al. performed a label-free quantitative MS-based analysis of human plasma-derived exosomes and microparticles isolated from patients with abdominal aortic aneurysm and control subjects, to find potential circulating biomarkers of this pathology [132]. The authors found differential protein profiles in patients and some of these altered proteins were involved in important pathological mechanisms of abdominal aortic aneurysm progression such as oxidative stress, thrombosis and immune-inflammation. In patients, the average number of identified proteins in exosomes was 234, while in control subjects it was 210. In exosomes from patients, they identified a significant overexpression of ferritin, platelet factor 4, and C-reactive protein, that could be potential candidates for diagnosis, monitoring, or therapy of patients with abdominal aortic aneurysms. Even if this study was carried out on a small casistic, this data provided a preliminary list of proteins that contribute to characterizing the plasma proteome of patients with abdominal aortic aneurysms.

Kawasaki disease (KD) is a form of acute vasculitis that mainly affects children, where blood vessels become inflamed throughout the body. This pathology leads to the development of coronary artery aneurysms (CAA) that, in case of delay in treatment, can cause complications such as ruptures, thrombosis, stenosis, myocardial ischemia, or infarction. The treatment with an intravenous dose of immunoglobulin (IVIG) lowers the risk of CAA, even if it has been shown that up to 5% of treated patients still develop CAA compared to up to 25% of untreated children with KD [163]. Therefore, early detection and prompt treatment are crucial but, unfortunately, to date no specific laboratory tests are available for an early detection of CAA. A better understanding of the molecular pathogenesis of CAA could be useful to improve diagnosis and therapy in patients with KD. Recently, Xie et al. performed the first study of the protein profile of the serum exosomes of patients with CAA caused by KD [125]. Using 2-DE and MALDI-TOF/TOF MS they compared the proteomes of exosomes from samples collected from healthy children and children with CAA caused by KD. Even if it was a preliminary study on a small casistic, they demonstrated that levels of 32 exosomal proteins were significantly changed (18 upregulated and 14 downregulated) in patients with CAA compared to healthy controls, and these proteins were mainly involved in metabolic and immune system processes with several molecular functions among which the dominant were catalytic activity, enzyme regulator activity, and binding. Future studies will be necessary to clarify the molecular mechanisms of these proteins, but these preliminary results are a starting point that suggests an important role of exosomes in the pathogenesis of CAA caused by KD. In this context, another proteomic study has been carried out by Zhang et al. to investigate the global expression profile of serum exosomal proteins in patients with KD before and after the intravenous immunoglobulin therapy [164]. 2-DE coupled with MS analysis allowed the identification of 69 differentially regulated proteins between the two groups and 59 differential ones in patients after the therapy compared with the control subjects. From these, complement C3, apolipoprotein A-IV, and insulin-like growth factor-binding protein complex acid labile subunit displayed a modification in the expression before and after the therapy, indicating their potential role as biomarkers for monitoring therapy in patients with KD. Besides, most of the common proteins identified in both studies as altered in exosomes from KD patients are related to inflammation and CVD, and their clinical relevance will become the object of future research.

The protein profiling of exosomes isolated from serum samples of heart failure patients without allograft, heart transplant recipients with no rejection, with acute cellular rejection (ACR) and with antibody-mediated rejection (AMR), and control subjects were characterized for a non-invasive detection of cardiac allograft rejection [165]. LC-MS/MS analysis was performed on the different groups and 3537 proteins were identified, among which 45 proteins were able to distinguish the three patient groups revealed by principal component analysis (PCA). In particular, 17 differentially expressed exosomal proteins were found in the comparison of control, heart failure patients, and non-rejection heart transplant recipients, whereas 15 differently regulated proteins were measured in exosomes from non-rejection heart transplant recipients, ACR and AMR. Most of the identified proteins were involved in inflammation, complement activation, adaptive immunity, and coagulation. Therefore, this pilot study demonstrated that the proteome analysis of exosomes could be an effective non-invasive approach to diagnose and monitor acute transplant rejection through a panel of predictive and prognostic exosome-based biomarkers.

### 4.2. Exosomes Derived from Mesenchymal Stem Cell

A study on human embryonic stem cell-derived mesenchymal stem cell (MSC)-conditioned medium showed that MSC-generated exosomes reduced infarct size in a mouse model of myocardial ischemia/reperfusion injury [166]. Therefore, MSCs mediated cardioprotection by secreting exosomes. To understand the cardioprotective paracrine effects of MSCs, the authors fractionated the MSC-conditioned medium by ultrafiltration with membranes with different molecular weight cut off and demonstrated that the cardioprotective activity was performed by large complexes known as exosomes, which were then purified by size-exclusion fractionation on an HPLC. They reported an extensive list of MSC secreted proteins by LC-MS/MS analysis and many of these were exosome associated proteins such as CD9, CD81, ALIX, and superoxide dismutase 1 (SOD-1). In particular, they demonstrated that CD9 was a lipid membrane-bound protein while SOD-1 was localized within the lumen of exosome.

Similarly, in another study by Arslan et al., mice underwent 30 min ischemia followed by 24 h reperfusion, and purified exosomes or saline were administered intravenously before reperfusion, to evaluate the potential of MSC-derived exosomes to reduce cell death after myocardial ischemia/reperfusion injury in vivo [53]. Intact exosomes reduced infarct size by 45% compared to saline treatment, in a dose dependent manner, enhancing cardiomyocyte viability and cardiac function of the ischemic/reperfused myocardium. Besides, the exosome treatment increased ATP and NADH levels, decreased oxidative stress, induced phosphatidylinositol 3-kinase (PI3K)/protein kinase B (Akt) signalling, and reduced pro-apoptotic phosphorylation of c-JNK (c-Jun N-terminal Kinase). Cardiac and systemic inflammation were significantly reduced after reperfusion. Total protein levels were also evaluated by the authors but the two treatments, saline and exosomes, did not modify the protein content in the treated animals. Therefore, MSC-derived exosomes could be useful in the reperfusion therapy for AMI.

It is also well known that, the exosomal proteins derived from MSCs promote angiogenesis, decrease cardiomyocyte apoptosis and ventricular remodelling, and protect the cardiac function [151]. Ma et al. collected MSCs from human umbilical cords and evaluated whether exosomes released from Akt-overexpressing MSCs had a cardioprotection role and stimulated angiogenesis in AMI rat model [167]. They showed that the cardiac function was significantly improved after treatment with exosomes derived from Akt modified human umbilical cord-derived MSCs. Indeed, Akt-exosomes stimulated endothelial cell proliferation, migration and blood vessel formation, and showed a significant upregulation of the platelet-derived growth factor D (PDGF-D) expression level. Moreover, PDGF receptor β (PDGFR-β) protein expression was also increased in myocardial cells treated with Akt-exosomes. Therefore, PDGF-D may contribute in Akt-exosomes-mediated myocardial repair. The results obtained in this study are a starting point for developing a useful therapeutic strategy to improve cardiac function in patients after AMI.

Exosomes derived from MSC can also reduce myocardial ischemia/reperfusion injury by inducing cardiomyocyte autophagy via AMP-activated protein kinase (AMPK)/mTOR and Akt/mTOR pathways [168]. Rat cardiomyocytes were exposed in vitro to H_2_O_2_ and it was observed that H_2_O_2_ induced a dose-dependent increase of reactive oxygen species (ROS) production and cell apoptosis, whereas the autophagy gradually decreased with increasing hours from exposure. Apoptosis-related proteins and signalling pathway-related proteins were detected by Western blot analysis. The authors demonstrated that treatment with MSC-derived exosomes reduced H_2_O_2_-induced ROS production and cell apoptosis, and improved autophagy via the AMPK/mTOR and Akt/mTOR pathways. Therefore, the increased autophagy could represent a useful cytoprotective mechanism for cells after H_2_O_2_ stimulation. Moreover, in vivo, exosome injections in rats that underwent myocardial ischemia/reperfusion injury significantly reduced myocardial infarct size, improved myocardial viability and cardiac function. This study highlights the central role of autophagy in the beneficial action of MSC-derived exosomes on myocardial ischemia/reperfusion injury.

Some years ago, a study was also performed to comprehensively characterize the protein content of bone marrow derived MSCs and MSC-derived exosomes from cells cultured under normoxic conditions and under ischemic tissue simulated conditions using high-resolution isoelectric focusing coupled LC-MS/MS [169]. The authors identified and quantified a total of 6342 proteins in MSCs and 1927 proteins in MSC-derived exosomes, 457 of which were not detected in MSCs, demonstrating the exosomal enrichment. MSC-derived exosomes contained several angiogenic paracrine effectors that have increased expression level in MSCs exposed to ischemic tissue-simulated conditions. These factors included fibroblast growth factor, PDGF, epidermal growth factor, and nuclear factor-kappa B (NFkB) signalling pathway proteins. In particular, MSC-derived exosomes stimulated the angiogenesis in vitro in a dose dependent manner via the NFkB pathway in endothelial cells, showing that NFkB is an important mediator of angiogenesis. All these exosome-derived angiogenic paracrine effectors could be potential targets for the treatment of ischemic tissue-related diseases, such as peripheral arterial disease (PAD).

### 4.3. Exosomes Derived from Cardiac Fibroblasts

Cardiac fibroblasts (CFs) represent the most abundant non-myocyte subpopulation of cells in the heart, and they are involved in extracellular matrix (ECM) synthesis and turnover both in physiological and pathophysiological conditions. CFs are also able to secrete cytokines, growth factors and other signalling molecules that can affect all cardiac cell types and are involved in the response to a wide range of different stimuli during cardiac development, homeostasis, and disease. In a pathological condition, CFs are responsible for an excessive accumulation of ECM which, consequently, alters cardiac function leading to cardiac remodelling and heart failure. To date, however, few studies have investigated the composition and roles of exosomes derived from CFs. Cosme et al., for example, performed a proteomic analysis of mouse CF secretome, whole-CF lysate, and CF exosome content in normal and stressed conditions [170]. Differences in the number and content of the CF exosome proteins have been reported under normoxic and hypoxic conditions. Moreover, 144 proteins were significantly differentially expressed in exosomes between the two conditions. Comparing the exosome proteins, they also observed an increased expression of ECM proteins under hypoxia, as well as an overrepresentation of mitochondria-associated proteins, suggesting that hypoxic stress promotes mitochondria dysfunction and exosomes have an important role in the removal of dysfunctional mitochondria from the cell. Besides, they demonstrated that CF exosomes improved cardiomyocyte viability before the hypoxia treatment, whereas they reduced cardiomyocytes viability if added in the reoxygenation phase.

Another proteomic study demonstrated that angiotensin II (Ang II) increased exosome release from cultured neonatal rat CFs that, in turn, upregulated the expression levels of renin angiotensin system (RAS) in cardiomyocytes and downregulated angiotensin-converting enzyme 2 [171]. The CF-derived exosomes also stimulated Ang II production in cultured cardiomyocytes, thus amplifying the pathological cardiomyocyte hypertrophy via activating Ang II receptor type 1 (AT1R) and 2 (AT2R). Ang II-induced cardiac hypertrophy was inhibited by suppression of the exosome production by both AT1R and AT2R antagonists. Moreover, the authors performed a quantitative proteomic analysis of the exosomes released from CFs with or without Ang II stimulation, reporting that Ang II modifies the expression of a few CF exosome proteins involved in the regulation of PI3K/Akt and MAPKs pathways that mediate the upregulation of RAS leading to cardiomyocyte hypertrophy. Thus, this study demonstrated the paracrine mechanism between CFs and cardiomyocytes and the specific targeting of Ang II-induced exosome release from CFs could be a potential therapeutic strategy for the treatment of cardiac pathological hypertrophy and heart failure.

### 4.4. Exosomes Derived from Endothelial Cells

In the same way, ECs mediate the response to stress or damage signals not only through the release of cytokines and growth factors but also by exosomes that communicate with other cardiac cells [143]. De Jong et al. performed an iTRAQ-based quantitative proteomic analysis of the exosomes produced by cultured ECs exposed to different types of cellular stress to evaluate the effects of stress on protein content [39]. They identified 1354 proteins among which many were significantly altered in abundance after exposure to cellular stress, even if the protein levels in EC exosomes depended on the type of stress and culture condition suggesting a role of endothelial exosomes in the transfer of stress signals to the other cells. Moreover, ECs secrete exosomes to communicate with each other, for example to regulate angiogenesis. Indeed, a study demonstrated by a label-free quantitative MS/MS analysis that delta-like 4 (Dll4), a Notch ligand usually overexpressed during angiogenesis, was incorporated into human endothelial exosomes, and that these exosomes moved Dll4 protein to other ECs, resulting in the promotion of angiogenesis by inhibiting Notch signalling [172].

### 4.5. Exosomes Derived from Vascular Smooth Muscle Cells

A proteomic profiling of the exosomes from vascular smooth muscle cells (VSMCs) was also performed to better understand the communication between VSMCs and ECs that is important for the regulation of vascular development and homeostasis [173]. Exosomes are directly involved in this crosstalk. Proteins in the human VSMC-derived exosomes were identified using nanoLC-MS/MS after an in-gel digestion and subjected to gene ontology analysis. The authors identified 459 proteins in the VSMC-derived exosomes, mainly involved in cell-cell adhesion and platelet activation/coagulation. Therefore, the study suggested that the major functions of VSMC-derived exosomes are to maintain vessel homeostasis and regulate haemostasis and thrombosis, and the alteration of these regulatory functions contributes to the development of vascular diseases such as atherosclerosis, vascular calcification or aneurysm.

Vascular calcification is a frequent complication of atherosclerosis, chronic kidney disease, and diabetes mellitus. VSMCs promote vascular calcification which is a risk factor for cardiovascular morbidity and mortality. A proteomic study performed by Kapustin et al. demonstrated, for the first time, that VSMC calcification is mediated by regulated exosome secretion [174]. The authors employed the circulating calcification inhibitor fetuin-A to identify the origin of matrix vesicles, that are specialized membrane-bound vesicles secreted by VSMCs and responsible for initiation of calcification and loading of calcification inhibitors. They showed that fetuin-A accumulated in intracellular vesicles and colocalized with CD63 and lysosomal associated membrane protein (LAMP)-1/2, suggesting that it is trafficked and released via the exosome pathway, and thus identifying matrix vesicles as exosomal in origin. VSMC-derived exosomes were enriched with the tetraspanins CD9 and CD63, and their secretion was regulated by sphingomyelin phosphodiesterase 3. Indeed, the inhibition of sphingomyelin phosphodiesterase 3 blocked VSMC calcification, whereas the exosome secretion by VSMCs was increased in response to extracellular calcium and factors promoting calcification. Using LC-MS/MS the authors identified a total of 345 proteins in the VSMC-derived exosomes and performed a comparative proteomic analysis revealing that VSMC-derived exosomes had a protein composition similar to osteoblast-derived matrix vesicles. VSMC-derived exosomes mainly contained proteins involved in bone development, calcification, cell migration, and adhesion. Therefore, VSMC-derived exosomes are key players in calcification and vascular repair, representing potential therapeutic targets for prevention of myocardial infarction and CAD.

The same authors in a subsequent study investigated whether coagulation proteins also play a role in calcification [175]. VSMCs are protected from calcification by several inhibitors among which the vitamin K-dependent coagulation proteins (e.g., prothrombin (PT)). Circulating vitamin K-dependent coagulation proteins were measured in VSMC-derived exosomes, and their levels were increased under calcifying conditions. Exosomes were isolated from VSMCs treated in the absence or presence of PT, and it was found that PT inhibited exosome-induced calcification and reduced VSMC apoptosis. PT accumulated at sites of calcification, and its circulation level was reduced in patients with vascular calcification. Besides, the authors observed by immunohistochemistry that PT and fetuin-A colocalized in the vessel wall confirming that PT deposition occurs via exosomes and is stimulated by calcium, which is, for example, elevated in atherosclerotic plaques. The authors also demonstrated that VSMC exosomes had a thrombogenic activity in a tissue factor- and phosphatidylserine-dependent manner.

### 4.6. Exosomes Derived from Cardiac-Derived Progenitor Cells

The adult heart also contains a group of heterologous and multipotent cells known as cardiac-derived progenitor cells (CPCs) that are involved in heart and repair cardiac homeostasis. In culture, these cells can form spherical aggregates, named cardiosphere-derived cells (CDCs), which are a cardiac stem cell population with cardiac regenerative properties useful to repair the heart function in several cardiac diseases. CDC-derived exosomes reduce cardiac fibrosis and cardiomyocyte apoptosis post MI.

A few years ago, Barile et al. performed a label-free proteomic analysis of exosome derived from CPCs and bone marrow-derived mesenchymal stem/progenitor cells (BMCs) obtained from cardiac atrial appendage specimens and sternal bone marrow, respectively, of patients undergoing heart surgery for aortic valve disease, CAD, or both [176]. They identified numerous proteins significantly expressed at higher levels in CPC exosomes compared to BMC-secreted exosomes, and they reported pregnancy associated plasma protein-A (PAPP-A) as one of the most highly enriched proteins in CPC exosomes. CPC-secreted exosomes showed a cardioprotective capacity, enhancing ventricular function after ischemia/reperfusion, reducing scar size and improving ventricular function after permanent coronary occlusion in rats, and finally stimulating blood vessel formation. However, the cardioprotective role of exosomes derived from CPCs could also be due to PAPP-A, because this protein in its active form cleaved insulin-like growth factor binding protein-4 (IGFBP-4) promoting the release of insulin-like growth factor-1 (IGF-1) that is a key cardioprotective factor.

## 5. Conclusions and Future Perspectives

Extracellular vesicles are important bioactive mediators in intercellular communication, with a critical role in both physiological and pathological conditions. Nowadays, EVs are considered a promising source for biomarker discovery in several diseases and therapeutics. In particular, exosomes have recently emerged as an attractive target for diagnostic and therapeutic purposes in the field of cardiovascular diseases. Exosomes have aroused great interest in the biomedical field for investigating their functional roles in different pathophysiological conditions and for characterizing their protein content that always changes and adapts to local and external stimuli.

EVs are particularly abundant in bodily fluids, thus they can potentially be used in minimally invasive liquid biopsies that are able to provide information on patient diagnosis, improve prognosis, and define the follow-up protocol. The specific molecular signature of EVs could represent a picture of a specific disease in a liquid biopsy, encouraging the application of EVs to diagnose, or monitoring a pathological condition. EVs can be detected in blood, cerebrospinal fluid, and other biofluids of patients, and their genetic and proteomic content can reflect the cell of origin, thus constituting a disease-specific material. Therefore, there is a growing interest in using EVs as a platform for liquid biopsy-based biomarkers for disease diagnosis and therapeutic monitoring. EV platform for “liquid biopsy” is promising because it allows overcoming the invasiveness of tissue biopsy with a fast and high-throughput approach that is crucial in further clinical applications, and the circulating biomarkers in the body fluid reflect the changes in real time caused by tumours or a specific disease. Besides, EVs are stable particles released in circulation from several types of injured, stressed, or diseased cells, and their content is protected within the lipid membrane. Anyway, there are some challenges that should be resolved before the translation in clinics. Biofluids have an inherent biologic complexity and particular attention should be given to the optimization and standardization of the EV method of isolation that could significantly influence the quality and quantity of EVs collected. In addition, biological fluids have a dynamic nature and EVs collected from a biofluid are subject to changes related to the collection time and many physiological and pathological factors. In future, the validation with larger sample cohorts and prospective clinical trials will also be important to confirm the great potential of EVs for the liquid biopsy as a tool for disease diagnosis and therapeutic monitoring.

Many studies find that exosomes may play a pivotal role in cardiovascular field because they regulate the cardiac homeostasis and they are involved in a wide range of cardiovascular processes as signalling mediators in improving or worsening cardiac health. Different cardiac cells produce exosomes with various protein contents which have been studied in recent years to define a distinctive proteome that could be used as a potential diagnostic or prognostic biomarker for CVDs.

Several analytical methods have been developed to characterize EV protein contents and MS-based technologies made a significant contribution to providing a comprehensive and quantitative view of their proteome for diagnostic, prognostic, or therapeutic studies. However, further effort should be made to improve the isolation efficiency of exosomes to obtain high purity and recovery for the efficient evaluation of proteomic analysis. Variations of exosome isolation and characterization methods lead to a high variability in the exosomal protein content making it more difficult to define a single unique signature for a physiological or specific disease condition. The lack of standardized isolation and analysis methods is a key issue that should be resolved in future to minimize sources of preanalytical variability and to facilitate easier identification of valuable biomarkers. High throughput and standardized methods for preparation and analysis of exosome will ensure simple and fast measurements of exosomes in clinical settings.

**Figure 3:**
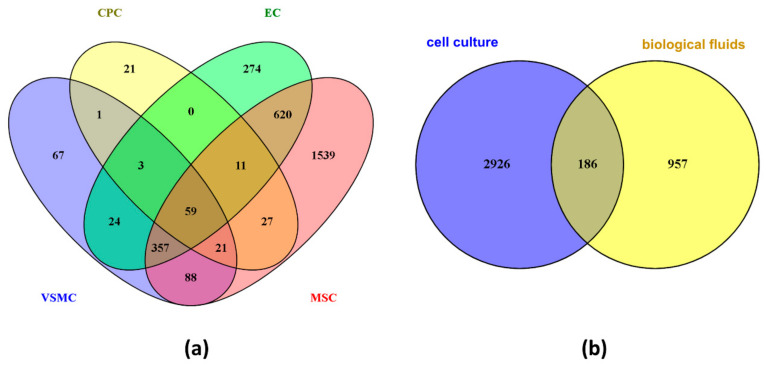
Venn diagram for exosome proteins identified in human studies. (**a**) Overlapping of proteins identified from different cell-derived exosomes. VSMC, vascular smooth muscle cells (blue); EC, endothelial cells (yellow); MSC, mesenchymal stem cells (green); CPC, cardiac derived progenitor stem cells (red). (**b**) Overlapping between identified proteins in exosome derived from cell cultures (blue) or from biological fluids (yellow).

In Figure 3, we showed the overlapping among the lists of proteins identified in exosomes derived from different cells in the human studies cited in this review. Despite the different number of totally identified proteins, due to the different technological approaches employed, it is however clear that, as expected, exosomes derived from different cells show a different protein cargo with specific proteins for each cell. On the other hand, the most impressive data is the low percentage of overlapping between exosomes isolated from cultured cells and those extracted from biological fluids, including serum, plasma, and pericardial fluid. In particular, those proteins identified only in exosomes from biological fluids are mainly involved in complement activation, immunoglobulin-mediated immune response, B cell receptor signalling pathway, positive regulation of lymphocyte activation, B cell activation or defence response, and are mainly located in the extracellular space (Panther overrepresentation test in the biological processes and cellular component categories, PANTHER version 15.0). Differently, analysing those proteins that are exclusively released in cell-derived exosomes, the mostly enriched biological processes are positive regulation of RNA polymerase II transcriptional preinitiation complex assembly, actin filament depolymerization, positive regulation of transcription elongation from RNA polymerase II promoter, endosome transport via multivesicular body sorting pathway, or ubiquitin-dependent protein catabolic process via the multivesicular body sorting pathway, with many intracellularly located proteins.

These data clearly suggest that there is still a lot to do in the standardization of isolation procedures in order to use exosome as biomarker, and that in vitro and in vivo studies could give different levels of information regarding exosome functions.

Extracellular vesicles show great potential in nanomedicine as noninvasively diagnostic and prognostic biomarkers, nanosized drug-delivery vectors, and therapeutic mediators in regenerative medicine.

The majority of clinical data on nanosized EVs as disease biomarkers have been obtained from cancer patients, but interesting data are emerging from studies on other diseases, such as CVDs. Moreover, EVs have also been used as vaccines in antitumor therapy and against infectious diseases, in preclinical trials, demonstrating feasibility and safety.

Stem cell therapy has proven to be an effective approach for CVDs treatment. However, there are several limitations, such as immunological compatibility, poor implantation and survival, or tumour formation, to be considered. In this respect, it has been shown that stem cell-derived exosomes have a myocardial protection function, thus representing an effective therapy for CVDs. Indeed, exosomes derived from stem cells are able to promote angiogenesis, cardiomyocyte proliferation and survival, and inhibit fibrosis and apoptosis.

Certainly, exosome therapy reduces the formation of any endogenous abnormal differentiation and adverse reactions (e.g., fever and allergic reactions) compared to stem cell transplantation, even if some challenges often limit the application of exosomes because it is difficult to determine their specific cell source, their extraction methods are not yet standardized, their amount is limited, and content is variable based on the pathophysiological conditions. In addition, highest purity of exosomes is required to increase their potential for clinical application.

Anyway, exosomes are promising molecules for therapeutic approaches because they have several advantages compared to cells, being biocompatible, non-immunogenic, non-tumorigenic, and physiologically more stable in circulation. Besides, exosomes can be sterilized by filtration, and handled and stored more easily than cells. In cardiovascular system, exosomes originate from different cardiac cell types in different conditions and contain interesting protein candidates that could generate positive or negative effects on the surrounding target cells. Therefore, stem cell-derived exosomes offers a promising cell-free approach in the future regenerative medicine.

Exosomes have a rapid hepatic clearance, thus recent studies have tried to ameliorate their accumulation and targetability to diseased sites in the cardiovascular system by surface functionalization. More attention should also be paid to the exosome production by engineering approaches, in particular to what the correct doses of exosomes reaching disease sites minimizing the side effects are and avoiding possible toxicity at high concentrations. Besides, to extend exosomal therapeutic potential, new techniques for loading non-native protein cargoes continue to be studied in the last few years. The application of these approaches in cardiovascular diseases is still at the beginning, but interesting and promising results have already been obtained in other pathologies such as cancer.

All these research studies will provide novel opportunities to use exosomes as diagnostic markers or to develop exosome-based therapeutic solutions that can be translated in the future into clinical practice for CVD management.

The future clinical use of EVs depends on the coordinated efforts in the field and for this purpose, recently, a consortium of scientists, clinical and industry partners founded the H2020 European Cooperation in Science and Technology (COST) program European Network on Microvesicles and Exosomes in Health and Disease (ME-HAD) with the aim to demonstrate the high potential of EVs for diagnosis, prognosis, and therapy in nanomedicine. They promote multidisciplinary approaches to studying EVs for future clinical applications as disease biomarkers or vectors of potential therapeutic molecules. The use of EVs in nanomedicine will pave the way for new protocols of personalized medicine, mostly in early clinical stages and as carrier systems for targeted drug delivery to a specific disease site or cell type. In the future it will be necessary to have a “gold standard” isolation and characterization method of EVs, as well as a repository of their proteome to increase the knowledge of potential predictive disease-related biomarkers and targets for therapy.

In conclusion, EVs have a significant clinical potential for diagnosis, prognosis, and therapy in the emergent precision and regenerative medicine.

## Figures and Tables

**Table 1:** Main characteristics of all EVs and the different subclasses.

			EV Subclasses	
	EVs *	Exosomes	Microvesicles	Apoptotic Bodies
DIAMETER	Small EVs: <100 or <200 nm; Medium or large EVs: >200 nm	30–150 nm	50–1000 nm and more	500–4000 nm
FLOATING DENSITY	Low density; Middle density; High density	Overlap in exosome and microvesicle density (1.08–1.19 g/mL) makes it difficult to distinguish the two groups [8]		1.16–1.28 g/mL [9]
BIOGENESIS	Naturally released from the cells. The EV assignment to a particular biogenesis pathway is sometimes difficult unless the use of live imaging techniques.	Multivesicular endosome	Plasma membrane budding and cleavage	Blebs detaching from dying apoptotic cell
MARKERS	Transmembrane or GPI-anchored proteins: non-tissue specific (tetraspanins, MHC class I, integrins, TfR2, LAMP1/2, heparan sulphate proteoglycans, EMMPRIN, ADAM10, NT5E, complement-binding proteins CD55 and CD59, SHH) and cell/tissue specific (e.g., glycophorin A, AChE-E, amyloid beta A4/APP) Cytosolic proteins: with lipid or membrane protein-binding ability (ESCRT-I/II/III, ALIX, flotillins-1 and 2, caveolins, annexins, heat shock proteins, syntenin) and promiscuous incorporation in EVs (HSP70, actin, tubulin, GAPDH).	ESCRT complex, tetraspanins (CD63, CD9, CD81, and CD82), flotillins, TSG101, ALIX, heat shock proteins (HSC70, HSP60, HSP70, HSPA5, CCT2, and HSP90)	Annexin A1, annexin V, flotillin-2, selectins, integrins, CD40	Phosphatidylserine, histones, annexin V, TSP, and C3b

This table meets the minimal information for studies of extracellular vesicles (MISEV) 2018 guidelines for the description of the protein markers that can be applied to all EVs [10]. Although MISEV 2018 does not propose markers that can specifically characterize each EV subtype, the table highlights the growing recognition of the existence of different EV types and reports all specific proteins that are mentioned in the most recent studies. AChE, acetylcholinesterase; ADAM10, disintegrin and metalloproteinase domain-containing protein 10; ALIX, programmed cell death 6-interacting protein; APP, amyloid precursor protein; CCT2, chaperonin containing TCP1 subunit 2; EMMPRIN, extracellular matrix metalloproteinase inducer; ESCRT, endosomal sorting complex required for transport; GAPDH, glyceraldehyde-3-phosphate dehydrogenase; HSC70, heat shock cognate 71kDa protein; LAMP1/2, lysosome associated membrane protein-1/2; MHC, major histocompatibility complex; NT5E, ecto-5′-nucleotidase or CD73; SHH, sonic hedgehog; TfR2, transferrin receptor 2; TSP, thrombospondin. * indicates MISEV 2018 guidelines.

**Table 2:** Scheme of the advantages and disadvantages of the main methods to EV isolation.

Isolation Techniques	Advantages	Limitations
Differential ultracentrifugation	Most commonly used method. Relatively simple method.	Time-consuming (ultracentrifugation steps, filtration and washing steps). Expensive equipment. Requires large sample volumes. Large number of samples cannot be processed simultaneously. The isolation efficiency can be influenced by multiple parameters (e.g., centrifuge rotor types, centrifugal force, and solution viscosity). Low recovery. Limited use in clinical practice.
Density-Gradient Ultracentrifugation	Provides high-purity exosomes.	Laborious procedure with a possible pre-analytical variability. Lack of process standardization. Provides relatively low recovery. Limited use in clinical practice.
Size-based isolation	Provides high yield. Provides effective separation with a minimal eluate volume. Relatively rapid and simple method. Inexpensive.	Removal of protein contaminants is not always ensured. Not suitable for the analysis of large sample volumes.
Immunoaffinity isolation	Highly selective isolation of exosomes. Reliability, reproducibility and rapidity of the analysis. Potential clinical routine application.	Success of the analysis strongly depends on the functional activity of antibodies. Expensive. Low yield.
Polymer-induced precipitation	Rapid, simple and high-throughput isolation of EVs. Inexpensive Simultaneous analysis of multiple samples. Small sample volumes. Preservation of bioactivity.	Possible presence of protein aggregates and coprecipitation of contaminants (e.g., lipoproteins and albumin) Precipitating polymer material is not compatible with the subsequent MS analysis

## Abbreviations

2-DE	Two-dimensional gel electrophoresis
ACR	Acute cellular reaction
AF4	Asymmetric flow field-flow fractionation
Akt	Protein kinase B
ALIX	Programmed cell death 6-interacting protein
AMI	Acute myocardial infarction
AMPK	AMP-activated protein kinase
AMR	Antibody-mediated reaction
Ang II	Angiotensin II
AT1R	Ang II receptor type 1
AT2R	Ang II receptor type 1
BMC	Bone marrow-derived mesenchymal stem/progenitor cell
CAA	Coronary artery aneurism
CAD	Coronary artery disease
CDC	Cardiosphere-derived cell
CF	Cardiac fibroblast
CHAPS	3-((3-cholamidopropyl)dimethylammonio)-1-propanesulfonic
c-JNK	c-Jun N-terminal kinase
CPC	Cardiac-derived progenitor cell
CVD	Cardiovascular disease
DDA	Data dependent acquisition
DIA	Data-independent acquisition
Dll4	Delta-like 4
DTT	Dithiothreitol
DU	De-ubiquitylating enzymes
EC	Endothelial cell
ECM	Extracellular matrix
EMT	Epithelial-to-mesenchymal transition
ERK	Extracellular signal-regulated kinase
ESCRT	Endosomal sorting complex required
EV	Extracellular vesicle
FASP	Filter aided sample preparation approach
FFF	Field flow fractionation
GPCR	G protein-coupled receptor
GPI	Glycosylphosphatidylinositol
HIF-1α	Hypoxia inducible factor 1α
HRS	Hepatocyte growth factor regulated tyrosine kinase substrate
HSC	Heat shock cognate
HSP	Heat shock protein
IGF1	Insulin-like growth factor 1
IGFBP-4	Insulin-like growth factor binding protein 4
IL-1β	Interleukin 1β
ILV	Intraluminal vesicle
IMAC	Immobilized metal ion affinity chromatography
iTRAQ	Isobaric tags for relative and absolute quantitation
IVIG	Intravenous dose of immunoglobulin
KD	Kawasaki disease
LAMP 1/2	Lysosomal associated membrane protein 1/2
LC	Liquid chromatography
MAPK	Mitogen-activated protein kinase
MED	Multiple enzyme digestion
MI	Myocardial Infarction
MRM	Multiple reaction monitoring
MS	Mass spectrometry
MSC	Mesenchymal stem cell
MV	Microvesicle
MVB	Multivesicular body
NFkB	Nuclear factor-kappa B
PAD	Peripheral arterial disease
PAPP-A	Pregnancy associated plasma protein-A
PAR1	Protease-activated receptor 1
PBS	Phosphate buffered saline
PCA	Principal component analysis
PDGF-D	Platelet derived growth factor D
PDGFR-β	Platelet derived growth factor receptor β
PEG	Polyethylene glycol
PI3K	Phosphatidylinositol 3-kinase
PRM-MS	Parallel reaction monitoring-mass spectrometry
PROMIS-Quan	Proteomics of microparticles using super-SILAC quantification
PT	Prothrombin
PUC-ERLIC	Prolonged ultracentrifugation-electrostatic repulsion-hydrophilic interaction chromatography
RAS	Renin angiotensin system
ROCK	RHO associated protein kinase
ROS	Reactive oxygen species
SDS	Sodium dodecyl sulphate
SEC	Size exclusion chromatography
SILAC	Stable isotope labelling with amino acids in cell culture
SNARE	Soluble N-ethylmaleimide-sensitive fusion protein attachment protein receptor
SOD-1	Superoxide dismutase 1
STAM	Signal-transducing adaptor molecule
SWATH	Sequential window acquisition of all theoretical fragment ion
TFF	Tangential flow filtration
TiO2	Titanium dioxide
TLR	Toll like receptor
TNF-α	Tissue necrosis factor α
VPS4	Vacuolar protein sorting-associated protein 4
VSMC	Vascular smooth muscle cell
VTA1	Vacuolar protein sorting-associated protein VTA1 homolog
